# Hip Muscle Strength Explains Only 11% of the Improvement in HAGOS With an Intersegmental Approach to Successful Rehabilitation of Athletic Groin Pain

**DOI:** 10.1177/03635465211028981

**Published:** 2021-08-16

**Authors:** Samuel R. Baida, Enda King, Chris Richter, Shane Gore, Andrew Franklyn-Miller, Kieran Moran

**Affiliations:** † Sports Medicine Department, Sports Surgery Clinic, Dublin, Ireland; ‡ School of Health and Human Performance, Dublin City University, Dublin, Ireland; § Insight Centre for Data Analytics, Dublin City University, Dublin, Ireland; ‖ Department of Life Sciences, Roehampton University, London, UK; ¶ Centre for Health, Exercise and Sports Medicine, University of Melbourne, Melbourne, Australia; Investigation performed at Sports Surgery Clinic, Santry Demesne, Dublin, Ireland

**Keywords:** groin pain, hip strength, reactive strength, HAGOS

## Abstract

**Background:**

Exercise-based rehabilitation targeting intersegmental control has high success rates and fast recovery times in the management of athletic groin pain (AGP). The influence of this approach on hip strength and lower limb reactive strength and how these measures compare with uninjured athletes (CON) remain unknown. Additionally, the efficacy of this program after return to play (RTP) has not been examined.

**Purpose:**

First, to examine differences in isometric hip strength, reactive strength, and the Hip and Groin Outcome Score (HAGOS) between the AGP and CON cohorts and after rehabilitation; second, to examine the relationship between the change in HAGOS and the change in strength variables after rehabilitation; last, to track HAGOS for 6 months after RTP.

**Study Design:**

Cohort study; Level of evidence, 2.

**Methods:**

A total of 42 athletes diagnosed with AGP and 36 matched controls completed baseline testing: isometric hip strength, lower limb reactive strength, and HAGOS. After rehabilitation, athletes with AGP were retested, and HAGOS was collected at 3 and 6 months after RTP.

**Results:**

In total, 36 athletes with AGP completed the program with an RTP time of 9.8 ± 3.0 weeks (mean ± SD). At baseline, these athletes had significantly lower isometric hip strength (abduction, adduction, flexion, extension, external rotation: *d* = *–*0.67 to −1.20), single-leg reactive strength (*d = −*0.73), and HAGOS (*r = −*0.74 to −0.89) as compared with the CON cohort. Hip strength (*d* = −0.83 to −1.15) and reactive strength (*d = −*0.30) improved with rehabilitation and were no longer significantly different between groups at RTP. HAGOS improvements were maintained or improved in athletes with AGP up to 6 months after RTP, although some subscales remained significantly lower than the CON group (*r = −*0.35 to −0.51). Two linear regression features (hip abduction and external rotation) explained 11% of the variance in the HAGOS Sports and Recreation subscale.

**Conclusion:**

Athletes with AGP demonstrated isometric hip strength and reactive strength deficits that resolved after an intersegmental control rehabilitation program; however, improved hip strength explained only 11% of improvement in the Sports and Recreation subscale. HAGOS improvements after pain-free RTP were maintained at 6 months.

Athletic groin pain (AGP) is an overuse musculoskeletal presentation accounting for 9.4% of all injuries in male Gaelic football,^35^ with similar percentages in soccer^54^ and Australian rules football.^40^ The diagnosis encompasses the clinical presentation of pain at musculotendinous or fascial attachments to the anterior pelvis (eg, proximal adductor tendon, pubic aponeurosis, inguinal ligament, iliopsoas tendon).^12^ It can result in reduced athletic performance, sporting participation, and health-related quality of life.^37^ Exercise-based rehabilitation is effective in treating athletes with AGP when compared with passive^20^ or surgical^27^ interventions.

Nonsurgical rehabilitation of AGP has traditionally targeted the painful structures through hip- and trunk-strengthening programs aiming to increase the tissue’s capacity to tolerate additional load.^20,55^ A potential limitation to this approach may arise when determining which structure to rehabilitate when multiple pathologies exist, as is commonly found clinically in athletes with AGP.^19^ In addition, this approach may not address overall movement control, which may have contributed to the initial injury.^14^ More recent published research outlined an intervention program, inclusive of all AGP pathologies, which aimed to reduce overload on the injured structures/region by targeting intersegmental control of the trunk, pelvis, and hip through strength, running, and change-of-direction exercises.^26^ Intersegmental control describes the coordinated relationship between the trunk and lower limb segments (ie, hip, knee, and foot) to produce efficient multijoint, multiplanar movements, which cannot be evaluated via the assessment of singular muscle groups.^14^ When this intersegmental rehabilitation approach was utilized, the anatomical diagnosis of AGP did not influence the return-to-play (RTP) times, and overall this approach did demonstrate quicker RTP times when compared with studies using the traditional approach (mean RTP time, 9.9 vs 12.8-18.5 weeks).^20,55,57^ After intersegmental rehabilitation, significant trunk and lower limb kinematic and kinetic changes were also noted in change-of-direction technique, which were associated with improved change-of-direction performance.^26^ Furthermore, significant improvements were found in self-reported pain and function (as measured by the Hip and Groin Outcome Score [HAGOS]) and pain provocation tests (bilateral squeeze test in 0°, 45°, and 90° of hip flexion).^26^ It has been proposed that isometric hip strength and strength ratio measures be included in the assessment of AGP, as they have been reported as risk factors for groin injury.^10,45^ However, these measures have not been assessed before or after an intersegmental rehabilitation approach.

In addition to isometric hip strength, lower limb reactive strength^32^ and interlimb asymmetry^21^ have been associated with increased risk of lower limb injury and have not been examined in athletes with AGP. Reactive strength reflects an athlete’s explosive neuromuscular capacity utilizing the stretch-shortening cycle (ie, rapid change from eccentric to concentric muscular contraction) and has been quantified using the reactive strength index (RSI) during a drop jump.^13^ In athletes with AGP, longer ground contact times (GCTs) have been reported during plyometric actions when compared with uninjured controls,^15^ suggesting reduced reactive strength capacity.^29^

Interlimb asymmetry has been frequently used during rehabilitation to quantify the difference in strength or performance of 1 limb with respect to the other.^3^ Previous research has suggested that athletes with interlimb asymmetries >15% may have an increased risk of lower limb injury and thereby asymmetries can provide important markers for rehabilitation and RTP status.^21^ The importance of limb asymmetry in relation to AGP remains unknown. Further to the examination of strength measures (ie, hip strength, reactive strength), the contribution of changes in strength to improvements in HAGOS is unknown. For clinicians rehabilitating athletes with AGP, a greater understanding of how strength is related to recovery of sporting function may help enhance rehabilitation programs.

The long-term efficacy of rehabilitation is extremely important given the high reinjury rates reported in patients with AGP.^56^ In the return to sports after rehabilitation, the initial 6-month period is crucial, as the increasing physical demands placed on athletes during this period can increase susceptibility to reinjury of soft tissue structures.^4,39^ Assessment of HAGOS over this period can provide important insight into the efficacy of the intersegmental control after athletes resume full training and play over a longer period.

The primary aim of this study was to examine isometric hip strength (peak torque and peak torque ratios), reactive strength, interlimb asymmetry in isometric hip and reactive strength, and patient-reported outcomes (HAGOS, Marx Activity Rating Scale) in athletes with AGP from baseline (prerehabilitation) to RTP (postrehabilitation) and in comparison with uninjured athletes (control group; CON). A secondary aim was to examine the relationship between the pre- to postrehabilitation change in strength measures and the pre- to postrehabilitation change in HAGOS (Sports and Recreation subscale). The tertiary aim was to examine the changes in HAGOS subscales at 3 and 6 months after RTP after rehabilitation targeting intersegmental control.

The following was hypothesized: (1) isometric hip strength and reactive strength would be lower and interlimb asymmetries would be greater in the AGP cohort as compared with the CON cohort at baseline testing and would normalize to values observed in the CON cohort after rehabilitation; (2) a positive association would exist between the increase in strength measures and the increase in HAGOS Sports and Recreation subscale score after rehabilitation; and (3) HAGOS would improve in the AGP group at RTP and would be maintained at 6-month follow-up.

## Methods

This study was designed as a cohort study with a pre- to postintervention trial. The study was conducted in the sports medicine department of the Sports Surgery Clinic, Dublin, Ireland. Enrollment started June 2018 and ended October 2019, with the last follow-up in April 2020. Data collection was not affected by COVID-19, and the government advised activity restrictions that were put into place on March 27, 2020. Only 1 HAGOS 6-month follow-up was collected after this time, which was not influenced by these restriction measures. On the basis of previous HAGOS data,^26^ with 80% power and an alpha error probability of .05, 36 participants were required a priori. To facilitate a potential dropout rate of 15%,^26^ 42 participants were recruited.

### Eligibility Criteria

A clinical diagnosis was determined by a sports and exercise medicine physician (A.F.M.) after a directed history, clinical examination, and review of magnetic resonance imaging findings as previously described.^12^ Inclusion criteria included the following: anatomic diagnosis falling under AGP (iliopsoas, adductor, pubic aponeurosis, inguinal, and hip),^12^ men aged 18 to 35 years involved in multidirectional field-based sports, hip/groin symptoms during sporting activity with duration >4 weeks, and plan to return to same preinjury sport and level of competition. Exclusion criteria were as follows: hip joint arthrosis (grade ≥3 on magnetic resonance imaging), an underlying medical condition (eg, inflammatory arthropathy or infection), and history of hip and/or groin surgery. Control participants were recruited via social media outlets and local sporting clubs and were matched according to age, sports played, and level of competition. Control participants were included if they had no previous groin or lower limb surgery and no lower limb injury within the previous 3 months. All participants provided informed consent. Ethical approval was granted by the Sports Surgery Clinic’s ethics boards (SAREB15/10/18 SB/CB).

### Protocol

Athletes attended the clinic for baseline testing (prerehabilitation) of clinical and strength-related outcome measures. Athletes with AGP repeated all testing at RTP after the rehabilitation program and completed the HAGOS and Marx questionnaires electronically at 3 and 6 months after RTP.

### Clinical Outcome Measures

RTP criteria have been defined^26^ and include symmetrical hip flexion/internal rotation range of motion and pain-free squeeze test in 45° and 0° of hip flexion,^9^ pubic stress test,^18^ and linear and multidirectional running.^26^ The squeeze tests were recorded using a sphygmomanometer (Welch Allyn), preinflated to 20 mm Hg, with a maximum value and a value at first onset of pain recorded.^9,18^ Self-reported disability and function were assessed using the HAGOS (0-100, with 100 indicating nil problems),^49^ and the level of sporting activity was assessed with the Marx activity scale^22^ (0-16, with higher scores indicating increased frequency of high-demand sporting activity).

### Strength-Related Outcome Measures

Hip strength was assessed with a handheld dynamometry (Commander JTECH) per a previously published protocol.^50^ Intratester and interday reliability was examined and confirmed before commencing this study (Appendix A1, available in the online version of this article). Both limbs were tested for all athletes, with the order standardized to ensure systematic performance: flexion-supine (FLEX), extension-prone (EXT), abduction−side lie (ABD), adduction–side lie (ADD), internal rotation−sitting (IR), and external rotation–sitting (ER). Strength ratios included ADD/ABD, EXT/FLEX, and ER/IR. The length of the lever arm was measured (distance between approximate axis of rotation and the point of the application of force) and used to calculate torque (lever arm length [m] × force [N]), and values were normalized to body mass (N·m/kg).^47^ To reduce potential systematic differences in test and retest results,^50^ the maximum value (from 4 trials) was used in the statistical analysis.

Lower limb reactive strength was assessed using a double- and single-leg drop jump (DLDJ and SLDJ). The protocol is outlined in Appendix A2 (available online). Two force plates (40 cm × 60 cm, 1000 Hz; BP400600 [AMTI]) were used to collect GCT. Jump height (JH) was calculated from flight time.^13^ RSI was calculated by dividing JH (centimeters) by GCT (seconds). The average of the 3 trials was used in the analysis.

### Intervention

**Figure 1.**
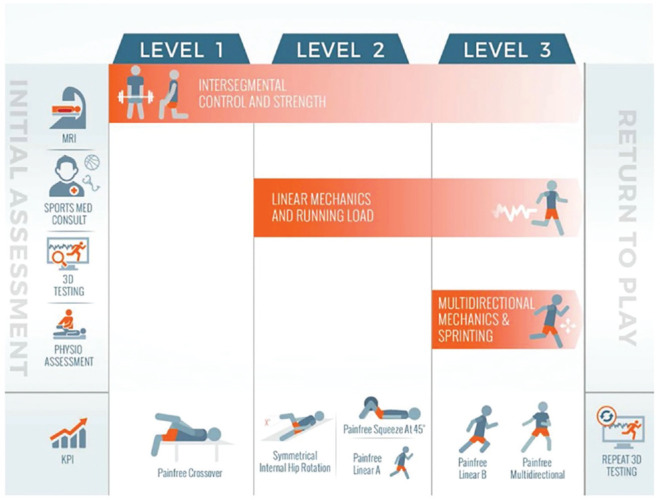
Overview of the intersegmental control program, including the 3 rehabilitation levels and the criteria to progress through the program and return to play. 3D, 3-dimensional; KPI, key performance indicators for progression; MRI, magnetic resonance imaging.

The intersegmental rehabilitation program focused on specific components of recovery (Figure 1), exercise selection, and progression based on athletes’ movement quality and competency, rather than being focused on strengthening specific muscles in individual planes per traditional rehabilitation approaches. The focus of intersegmental control was consistent throughout the 3 levels of the program (level 1, strength; level 2, linear running mechanics; level 3, change-of-direction mechanics) with exercise selection and coaching concentrating on improving movement patterns. The program was delivered by 3 experienced physical therapists (S.R.B., E.K.), with athletes attending supervised rehabilitation appointments approximately every 14 days depending on availability. Between supervised rehabilitation sessions, athletes trained unsupervised with level 1 exercises performed 4 times per week and run sessions performed 2 times per week. A detailed description of the program is presented in Appendix A3 (available online), which highlights a number of exercise modifications to the original intervention program^26^ by our research group to simplify coaching and for ease of implementation by athletes.

### Data Analysis

Data processing and descriptive statistics were carried out using MATLAB (Version R2015a; MathWorks). Isometric hip strength, reactive strength, bilateral squeeze test, and interlimb symmetry data are presented as mean and standard deviation; normality was assessed (Shapiro-Wilk test); and parametric statistics were applied. The asymmetry index was used to test interlimb symmetry,^44^ where *Sx* is the symptomatic limb and *Nx* is the nonsymptomatic limb:

asymmetry index = [(*Sx – Nx*) / (*Sx*+*Nx* / 2)] × 100.

The asymmetry index can provide both a magnitude (taking the absolute value of the interlimb difference) and a direction (maintaining the positive or negative sign, with a negative sign indicating a greater value on the nonsymptomatic limb). The magnitude of asymmetry was used to compare the AGP and CON cohorts, and the direction of asymmetry was used to compare athletes with AGP before and after rehabilitation.

To compare the AGP and CON cohorts, symptomatic limbs were matched to uninjured athletes based on limb dominance (self-selected as an athlete’s preferred kicking leg). To detect differences in athletes with AGP from baseline to RTP, paired *t* tests were used. Between the AGP and CON groups (AGP baseline vs CON; AGP RTP vs CON), independent *t* tests were used according to a per-protocol analysis. As the HAGOS data were not normally distributed, nonparametric statistics were applied. A Friedman analysis of variance with repeated measures was used to examine the HAGOS subscale scores in the AGP group across all 4 time points (baseline, RTP, and 3 and 6 months after RTP). Post hoc analysis (Wilcoxon signed-rank test) was used to independently compare the HAGOS time points pairwise. When HAGOS subscale scores were examined between the AGP and CON groups, Mann-Whitney *U* tests were applied. Significance was set at *P* < .05. Effect sizes for parametric tests were calculated according to Cohen *d*, with thresholds of small (<0.50), medium (0.50-0.80), and large (>0.80).^8^ For nonparametric tests, effect sizes (*r*) were calculated by dividing the *z* value by the √*N*, with thresholds of small (<0.1), medium (0.1-0.3), and large (>0.5).^41^

To more robustly examine the study aims and increase the generalizability of our findings, a permutation technique (with replacement) was applied.^6^ Briefly, 75% of the AGP cohort was selected from the data and statistically compared with 27 controls who were randomly matched for leg dominance. This process was repeated 100 times, with all random data sets condensed to their average values (*P* value and effect size) and the number of significant differences reported as a percentage. When the consistency of significant differences was ≥85%, the variable was reported as significant.

Pearson correlation coefficients were calculated to quantify the degree of relationship between self-reported sporting function (as measured by the pre- to postrehabilitation change in HAGOS Sports and Recreation subscale score) and hip strength and reactive strength (as measured by the pre- to postrehabilitation changes in peak isometric hip torque and DLDJ/SLDJ RSI, JH, GCT). Additionally, a linear regression analysis was applied with recursive feature elimination and a 5 × 3–fold nested cross-validation to examine the ability of the hip strength and RSI variables to predict the changes in the HAGOS Sports and Recreation subscale score.

## Results

**Figure 2.**
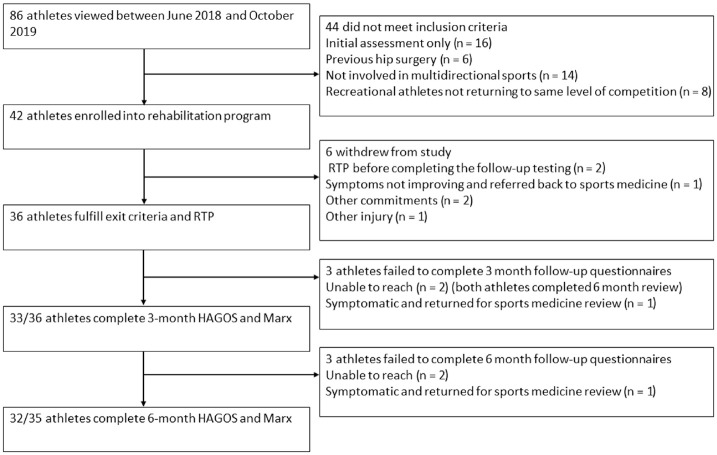
Participant flow through the study. HAGOS, Hip and Groin Outcome Score; Marx, Marx Activity Rating Scale; RTP, return to play.

The flow of athletes through the study is presented in Figure 2. In total, 86 athletes were referred for inclusion; 44 did not meet inclusion criteria; 42 enrolled in the study; and 6 withdrew before achieving the RTP criteria. Two athletes returned to play without completing the follow-up testing; 2 withdrew owing to other commitments; 1 remained symptomatic and was referred for review with the sports and exercise medicine physician; and 1 sustained a lower limb injury (work related). A total of 36 athletes met the RTP criteria in an average of 9.8 ± 3.0 weeks, and 4 athletes were lost to follow-up at 6 months after RTP. Two of the 4 athletes experienced recurrent symptoms and were reviewed by the sports and exercise medicine physician, and 2 were uncontactable. Athlete demographics are presented in Table 1, with no significant differences observed for age, height, or weight. The most common anatomic diagnoses were pain or tenderness at the pubic aponeurosis (57%), followed by proximal adductor tendon insertion (19%), iliopsoas (14%), hip (8%), and inguinal (2%). A second and tertiary diagnosis falling under the umbrella diagnosis of AGP was reported in 60% and 17% of athletes, respectively. Each athlete attended the clinic an average of 4.7 ± 1.3 appointments.

**Table 1:** Athlete Characteristics, Sports Played, and Clinical Diagnoses^*a*^

Group	AGP (n = 42)^*b*^	CON (n = 36)	*P* Value
Age, y	25.9 ± 4.9	24.1 ± 4.5	.169
Height, cm	1797.1 ± 64.5	1809.5 ± 57.8	.408
Mass, kg	80.3 ± 7.2	80.4 ± 8.2	.938
Sports played, %			
GAA football	58	67	
Soccer	25	17	
GAA hurling	14	6	
Rugby union	3	8	
Basketball	0	3	
Symptom duration, wk	38.7 ± 5.5	—	

a Values are presented as mean ± SD unless noted otherwise. AGP, athletic groin pain; CON, control; GAA, Gaelic Athletics Association; —, not relevant.

b Primary diagnoses: pubic aponeurosis, 57% (n = 24); adductor longus, 19% (n = 8); psoas, 14% (n = 6); hip, 8% (n = 3); and inguinal, 2% (n = 1).

### Clinical Outcome Measures: Baseline to RTP

Table 2 presents all HAGOS findings and between-group comparisons. All HAGOS subscale scores (*P* < .001; *r* = −0.74 to −0.89) and the Marx score (*P* < .001; *r* = *−*0.70) were significantly lower in the AGP cohort as compared with the CON cohort at baseline testing, with large effect sizes evident. After rehabilitation, all HAGOS subscale scores (*P* < .001; *r* = 0.50 to 0.60) and Marx score (*P* = .002; *r* = −0.42) demonstrated significant improvements of large effect in the athletes with AGP. At RTP, there was no difference in HAGOS Symptoms score (*P* = .112; *r* = −0.27) between the AGP and CON groups, while all other HAGOS outcomes (*P* < .02; *r* = −0.40 to −0.77) and Marx score (*P* = .001; *r* = −0.61) remained significantly lower, with medium to large effect size differences evident. At baseline, 72% of athletes with AGP reported pain during the squeeze test in 45° and 61% during the squeeze test in 0°; at RTP, no athletes indicated pain during either test (see Appendix 4, available online, for squeeze test values).

**Table 2:** HAGOS Subscale Scores: AGP Group (All Time Points) and Control Athletes^*a*^

	Median (IQR)					AGP (Baseline) vs CON		AGP (Baseline) vs AGP (RTP)		AGP (RTP) vs CON		AGP (RTP) vs AGP (3 mo)		AGP (3 mo) vs AGP (6 mo)		AGP (6 mo) vs CON	
HAGOS	CON	AGP Baseline^*b*^	AGP RTP^*c*^	AGP 3 mo	AGP 6 mo	*P* Value	*r*	*P* Value	*r*	*P* Value	*r*	*P* Value	*r*	*P* Value	*r*	*P* Value	*r*
Symptoms	89.3 (84.8-97.3)	60.2 (56.3-75.0)	83.9 (75.0-92.9)	89.3 (81.3-93.8)	85.7 (81.3-93.8)	<.001	−0.74	<.001	−0.58	.112	−0.27	.251	−0.14	.84	−0.03	.085	−0.21
Pain	97.5 (94.4-100.0)	76.3 (63.1-85.6)	92.5 (85.0-97.5)	96.3^*d*^ (87.5-97.5)	96.3^*e*^ (87.5-97.5)	<.001	−0.76	<.001	−0.53	.020	−0.40	.200	−0.16	.35	−0.12	.003	−0.36
ADL	100.0 (98.8-100.0)	75.0 (70.0-90.0)	95.0 (88.8-100.0)	100.0^*d*^ (88.8-100.0)	95.0^*e*^ (85.0-100.0)	<.001	−0.76	<.001	−0.50	.014	−0.41	.126	−0.19	.24	−0.15	.004	−0.35
Sports Rec	98.4 (93.9-100.0)	54.7 (39.9-67.2)	85.9 (80.5-93.8)	87.5^*d*^ (78.1-96.9)	87.5^*e*^ (77.4-94.5)	<.001	−0.82	<.001	−0.60	.002	−0.48	.613	−0.06	.86	−0.02	<.001	−0.48
PA	100.0 (100.0-100.0)	6.3 (0.0-37.5)	50.0 (21.9-75.0)	87.5^*d,f*^ (75.0-100.0)	93.8^*e*^ (75.0-100.0)	<.001	−0.89	<.001	−0.52	<.001	−0.77	<.001	−0.45	.64	−0.06	.002	−0.38
QOL	100.0 (90.0-100.0)	35.0 (30.0-45.0)	67.5 (45.0-80.0)	77.5^*d,f*^ (67.5-90.3)	80.0^*e*^ (68.8-95.0)	<.001	−0.83	<.001	−0.56	<.001	−0.69	.008	−0.32	.52	−0.08	<.001	−0.51
Marx	16.0 (13.8-16.0)	4.0 (0.0-8.3)	12.0 (9.0-12.0)	12.0 (11.8-16.0)	12.0 (8.7-12.3)	<.001	−0.70	.002	−0.42	<.001	−0.61	.099	−0.20	.67	−0.05	<.001	−0.48

a Effect size: *r* < 0.1 (small), *r* = 0.1 to 0.5 (medium), *r* > 0.5 (large). ADL, Activities of Daily Living; AGP, athletic groin pain; HAGOS, Hip and Groin Outcome Score; IQR, interquartile range; Marx, Marx Activity Scale; PA, Physical Activity; QOL, Quality of Life; RTP, return to play; Sport Rec, Sports and Recreation.

b Each value, *P* < .001: AGP baseline < CON.

c Each value, *P* < .001: AGP RTP > AGP baseline.

d *P* < .05: AGP 3 months < CON.

e *P* < .05: AGP 6 months < CON.

f *P* < .01: AGP 3 months > AGP RTP.

### HAGOS Follow-up at 3 and 6 Months After RTP

**Figure 3.**
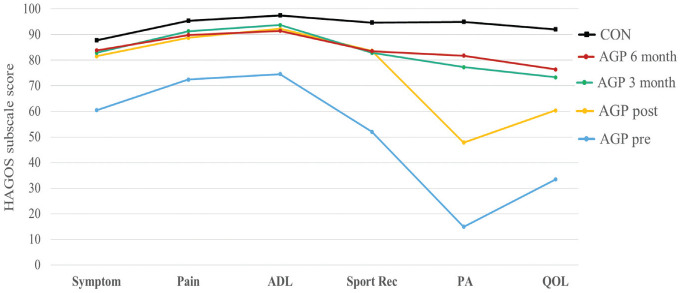
Hip and Groin Outcome Score (HAGOS) subscale scores for athletes across all time points: control (CON) and athletic groin pain (AGP). ADL, Activities of Daily Living; PA, Physical Activity; Post, postrehabilitation; Pre, prerehabilitation; QOL, Quality of Life.

The response rate for HAGOS and Marx questionnaires was 92% at 3 months and 91% at 6 months. From RTP to 3 months, HAGOS Physical Activity (*P* < .001; *r* = −0.45) and Quality of Life (*P* = .008; *r* = −0.32) significantly increased with medium effects, while all other HAGOS subscale and Marx scores were maintained. From 3 to 6 months after RTP, no significant changes were found in any HAGOS or Marx score, and at 6 months after RTP, no difference in HAGOS Symptoms scores was evident between the AGP and CON groups. However, HAGOS Pain, Activities of Daily Living, Sports and Recreation, Physical Activity, and Quality of Life subscale scores were significantly lower in the AGP cohort in comparison with the CON cohort, with differences of medium effect sizes evident (*P* < .003; *r* = −0.35 to −0.51) (Figure 3).

### Strength Outcome Measure: Baseline to RTP

**Figure 4.**
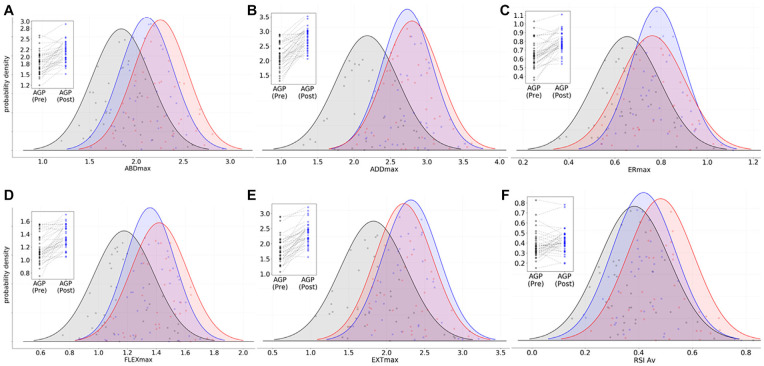
(A-E) Peak (max) isometric hip strength (N·m/kg) among muscle groups: abductors (ABD), adductors (ADD), external rotators (ER), flexors (FLEX), and extensors (EXT). (F) Average single-leg reactive strength index (RSI Av). Black shade, athletes in AGP group at baseline (PRE); blue shade, return-to-play (POST); red shade, uninjured control athletes (CON). Inset box: individual changes in athletes with AGP from baseline to return-to-play testing. AGP, athletic groin pain; Av, average; max, maximum; RSI, reactive strength index.

Five of the 6 peak hip torque measures (ABD: *P* < .001, *d* = −1.20; ADD: *P* < .001, *d* = −1.20; FLEX: *P* < .001, *d* = −1.07; EXT: *P* = .005, *d* = −0.83; ER: *P* = .03, *d* = −0.67) and SLDJ RSI (*P* = .014; *d* = −0.73) were significantly lower in the AGP group than the CON group at baseline testing, with differences of medium to large effect sizes evident. All 5 peak hip torque measures demonstrated significant increases in the AGP group after rehabilitation of large effect (*P* < .001; *d* = −0.83 to −1.15), while a small increase was evident in the SLDJ RSI (*P* = .093; *d* = *–*0.30). At RTP testing, no significant differences were found in any of these strength measures as compared with the CON group (Figure 4). No significant differences occurred in any hip torque ratios (ADD/ABD, EXT/FLEX, ER/IR) (Appendix A4, available online), DLDJ reactive strength measures (RSI, JH, GCT), or asymmetry index measures (peak isometric hip torque; SLDJ RSI, JH, CGT) (Appendix A5, available online) in any of the comparisons between the AGP and CON cohorts (ie, at baseline or at RTP) or in athletes with AGP from baseline to RTP. Full results for all isometric hip strength and reactive strength variables are presented in Appendix 4 (available online).

### Relationship Between Strength Outcome Measures and HAGOS Sports and Recreation

No significant correlations were found between the pre- to postrehabilitation change in HAGOS Sports and Recreation scores and the pre- to postrehabilitation change in strength outcome measures (peak isometric hip torque): ABD (*P* = .59; *r* = 0.32), FLEX (*P* = .104; *r* = 0.28), IR (*P* = .308; *r* = 0.18), EXT (*P* = .625; *r* = 0.08), ADD (*P* = .698; *r* = 0.07), ER (*P* = .561; *r* = −0.10), DLDJ (GCT: *P* = .90, *r* = 0.29; RSI: *P* = .160, *r* = 0.24; JH: *P* = .939, *r* = −0.01), and SLDJ (JH: *P* = .261, *r* = 0.21; RSI: *P* = .429, *r* = 0.14; GCT: *P* = .781, *r* = 0.05). The recursive feature elimination linear regression model selected 2 variables (pre- to postrehabilitation change in isometric hip ABD and hip ER torque) that could explain 11% of the variance in the change in HAGOS Sports and Recreation subscale score.

## Discussion

This study builds on previous research examining the efficacy of a rehabilitation program targeting intersegmental control among athletes with AGP, with similar RTP times (9.8 ± 3.0 weeks), RTP rates (86%), and significant changes of a large effect observed after rehabilitation per HAGOS.^15,26^ This is the first study to report HAGOS after RTP, and it found that athletes with AGP sustained or improved their HAGOS subscale scores over the 6 months after RTP. In addition, the findings highlighted a number of strength variables in the AGP cohort, including hip adduction strength, that were weaker at baseline testing with large differences in comparison with the CON group and that resolved after the rehabilitation program despite no specifically directed adductor-strengthening exercises. However, changes in hip and reactive strength explained only a small percentage of improvement in the HAGOS Sports and Recreation subscale score (11%), suggesting that other factors, such as intersegmental control, may hold greater importance in the rehabilitation of AGP.

The large improvements observed in all HAGOS subscale scores after AGP rehabilitation were larger than the smallest detectable change values previously reported for each subscale.^49^ The increase in HAGOS Physical Activity score from RTP to 3-month follow-up was also larger than the smallest detectable change. Notably, as the HAGOS Physical Activity score continued to improve over this period, indicating an increased level of physical activity, HAGOS Symptoms, Pain, and sporting function scores remained constant. This suggests improved capacity to tolerate the demands of sporting activities without reccurrence of pain. Furthermore, from 3 to 6 months after RTP, all HAGOS subscale scores and the Marx score remained constant in the AGP group, indicating that continued sporting participation did not negatively affect self-reported hip and/or groin function. At 6 months after RTP, all 6 HAGOS subscale scores in the AGP cohort had returned to the 95% reference range for hip and groin injury–free soccer players.^48^ Although similar to results from previous research, HAGOS remained lower when compared with the uninjured athletes with no history of hip and groin injury despite having made a pain-free RTP.^11,48^ In the current study, the lower HAGOS may be explained by the long duration of pain cited by athletes (mean, 39 weeks), as increased duration of pain (>6 weeks) has been shown to negatively affect all HAGOS outcomes.^51^

At baseline testing, athletes with AGP demonstrated large deficits in peak isometric hip torque in 5 of 6 muscle groups (ABD, ADD, FLEX, ER, and EXT torque) and medium deficits in SLDJ RSI when compared with the CON cohort. Previous research has shown comparable weakness of the hip ADD^38,47,52^ and ABD^43^ muscles in athletes with AGP when compared with uninjured controls, while no difference has been reported in hip FLEX,^42,47,52^ EXT,^33^ IR,^30^ or ER^30^ strength. Triplanar hip strength, particularly the hip extensors, abductors, and flexors, plays an important role in optimizing femoroacetabular control during single-leg activities^17,53^ during sports-specific movements. Insufficient strength to control the large external forces during such activities has been suggested to adversely affect movement technique^24^ and joint loading,^15^ resulting in excessive loading across the pubic symphysis.^7^ The lower SLDJ RSI observed in athletes with AGP was primarily due to longer GCT. This may represent reduced capacity to utilize the stretch-shortening cycle via a detraining mechanism^34^ attributed to injury, as normal sporting activity (eg, sprinting) can promote the stretch-shortening cycle function.^31^ Alternatively, the longer GCT may be due to athletes with AGP adopting a movement strategy to reduce the higher peak force and/or rate of loading that can occur with shorter ground contacts per the impulse momentum relationship.^5^ The increase in SLDJ RSI observed in the AGP cohort after rehabilitation resulted from a large effect size reduction in GCT. The shorter GCT may have been achieved through increased lower limb vertical stiffness^16^ and smaller angular displacements at the hip, knee, and ankle, although further biomechanical analysis is required. In athletes with AGP, training methods (eg, plyometrics) that utilize the stretch-shortening cycle and promote rapid expression of force in minimal times are of potential benefit during rehabilitation.

After successful rehabilitation, large increases were evident in all isometric hip strength variables and a medium increase in SLDJ RSI in the AGP group. No significant differences were evident in any of these variables at RTP when compared with the uninjured athletes. A major finding was that isometric adductor strength increased despite no specific adductor strength exercises being included in the intersegmental control program. Only 1 other AGP intervention study has objectively examined isometric hip strength (with handheld dynamometry) before and after rehabilitation.^57^ Yousefzadeh et al^57^ reported increased isometric hip adduction and abduction strength after rehabilitation, although the changes observed were larger than in the current study. This may be explained by the different intervention approaches employed, with Yousefzadeh et al utilizing exercises shown to induce high levels of adductor muscle activity (eg, Copenhagen adductor exercise).^46^ However, it is worth noting that the increase in hip adductor strength observed in our study was similar to the 35.7% increase in hip adductor strength that has been reported in uninjured soccer players after an 8-week program of targeted adductor muscle training.^23^ Two possible mechanisms may explain the increased adductor strength found in our study: first, reduced inhibition of the adductor muscle group as symptoms resolved with rehabilitation^28^; second, indirect muscle strengthening through the multiplanar action of the hip adductor musculature^36^ during the various levels of the rehabilitation program (eg, compound strength, linear run/change-of-direction exercises). No other study has examined the changes in hip flexion, extension, or external rotation strength after rehabilitation, and our findings (AGP baseline vs AGP RTP vs CON) highlight the potential importance of rehabilitation targeting an increase in triplanar hip strength in athletes with AGP. This is supported by the finding that only 11% of the pre- to postrehabilitation change in HAGOS Sports and Recreation subscale scores could be explained by 2 of the isometric peak torque measures (hip abduction and hip external rotation). This may suggest that factors other than isometric hip strength, such as intersegmental control through dynamic sporting actions (eg, running, change of direction), may play a more important role in explaining the improvements in HAGOS Sports and Recreation subscale scores after rehabilitation for AGP.

When muscle imbalances were examined between the AGP and CON groups at baseline testing, no significant differences were evident in any of the hip muscle strength ratios (ADD/ABD, EXT/FLEX, ER/IR) or any measure of interlimb asymmetry. This is consistent with the findings of Thorborg et al,^47^ who also reported no significant difference in hip ADD/ABD strength ratios when comparing soccer players with AGP and uninjured controls. Previous research has cited an ADD/ABD strength ratio <78% as a risk factor for groin injury^52^; however, we found ADD/ABD strength ratios >100% in the injured AGP group, indicating stronger adductor muscles relative to abductor muscles in athletes with AGP at baseline testing. Thus, targeting the ADD/ABD strength ratio for AGP rehabilitation would not appear relevant in this cohort of athletes.

In relation to interlimb symmetry, previous research has considered asymmetry indexes >15% as abnormal and therefore targets for rehabilitation.^1^ However, when examining athletes with AGP at baseline testing in our study, we found no asymmetry measures of isometric hip strength or reactive strength >6% favoring a particular limb. These findings suggest a bilateral reduction in isometric hip strength and reactive strength (given that significantly reduced strength measures were observed on the symptomatic limb); as such, rehabilitation may be enhanced by targeting both limbs rather than treating 1 side as symptomatic. Our hypothesis that there would be greater asymmetries in the injured population was rejected, which was an unexpected finding given our clinical experience and the asymmetries identified in lower limb injuries in other populations.^1,2,21,25^ There are a number of potential explanations for these findings. First, in our study 18% of participants with AGP cited bilateral symptoms; therefore, in these individuals there may be no preference to load or off-load a specific limb. Second, participants with AGP may have strength and movement deficits on the nonsymptomatic side that are driving or contributing to overload and pain on the symptomatic side. Last, the average asymmetry across a cohort may mask larger asymmetries in individual athletes. Given that there were strength deficits across all muscle groups at the hip, it is possible that some athletes had asymmetries in certain muscle groups but not in others, which may give the appearance of the absence of asymmetry in the cohort. Future research may be directed toward subgroup analysis of participants with AGP who consistently off-load a particular limb.

### Limitations

A true control group, undergoing no rehabilitation or sham treatment, was not utilized, and so it is unclear if there is a subset of athletes who improve without intervention. Uninjured athletes were tested only at baseline; therefore, it was not possible to assess the change in clinical and strength measures that occurred in uninjured athletes as they continued to participate in regular sporting activities. Adherence to the intersegmental rehabilitation program was not collected, and it is thus not possible to examine if all athletes completed the same volume and intensity of exercises prescribed. Female athletes were not included, and how our findings extrapolate to this athletic cohort remains unclear. HAGOS has been used to evaluate recovery after rehabilitation^26^ and has been advocated as part of the minimum reporting standards for clinical research on athletes with groin pain.^10^ In the current study, HAGOS data after RTP provided important information regarding self-perceived ability to perform specific tasks and activities; however, objective measures, such as hip strength and RSI, may be considered in future research to evaluate ongoing physical markers after RTP in athletes with AGP.

## Conclusion

In a cohort of athletes with AGP, rehabilitation targeting intersegmental control reproduced quick RTP times (vs programs targeting singular anatomic structures, 17.3 to 18.5 weeks)^20,55^ and confirmed significant improvements in all HAGOS subscale scores.^26^ HAGOS improvements were sustained (symptoms, pain, activities of daily living, sports and recreational function) and increased (physical activity and quality of life) up to 3 months after RTP, while all HAGOS improvements were then sustained up to 6 months. As compared with control, rehabilitation was effective at resolving the baseline deficits observed in single-leg reactive strength and isometric hip strength, including adductor strength despite the absence of targeted adductor strengthening. The strength measures had limited ability to explain the changes in HAGOS Sports and Recreation subscale scores, supporting the suggestion that other factors are important considerations in the rehabilitation of AGP, such as the targeting of intersegmental control during rehabilitation.

## Supplemental Material

sj-pdf-1-ajs-10.1177_03635465211028981 – Supplemental material for Hip Muscle Strength Explains Only 11% of the Improvement in HAGOS With an Intersegmental Approach to Successful Rehabilitation of Athletic Groin Pain Click here for additional data file. Supplemental material, sj-pdf-1-ajs-10.1177_03635465211028981 for Hip Muscle Strength Explains Only 11% of the Improvement in HAGOS With an Intersegmental Approach to Successful Rehabilitation of Athletic Groin Pain by Samuel R. Baida, Enda King, Chris Richter, Shane Gore, Andrew Franklyn-Miller and Kieran Moran in The American Journal of Sports Medicine
