# Supplementary material for: Hip Muscle Strength Explains Only 11% of the Improvement in HAGOS With an Intersegmental Approach to Successful Rehabilitation of Athletic Groin Pain
Source: Am J Sports Med. 2021 Aug 16;49(11):2994–3003. doi: 10.1177/03635465211028981 (PMC8411474; doi:10.1177/03635465211028981)
Supplement: sj-pdf-1-ajs-10.1177_03635465211028981 – Supplemental material for Hip Muscle Strength Explains Only 11% of the Improvement in HAGOS With an Intersegmental Approach to Successful Rehabilitation of Athletic Groin Pain [file sj-pdf-1-ajs-10.1177_03635465211028981.pdf]

On-line supplementary for: **Hip muscle strength explains only 11% of the improvement in HAGOS with an intersegmental approach to successful rehabilitation of athletic groin pain: Case control intervention with 6-month follow-up**

Appendix A1:

Intra-tester, inter-day reliability of peak isometric hip torque

Isometric hip strength was assessed with hand-held dynamometry (Commander J-tech) as per published protocol by Thorborg et al (2010).

Isometric hip strength examined: hip adduction (side-lie), abduction (side-lie), flexion (supine), extension (prone), internal and external rotation (seated)

N = 15 healthy participants with no previous history of AGP (8M/7F, age 23.6 ± 4.5years, height 1792 ± 53.4 cm, weight 76.1 ± 8.3 kg, Marx activity score 13.6 ± 2.5).

Intra-class correlation coefficient (ICC 2,1) ranged from 0.67 to 0.98, the standard error of measurement (SEM) ranged from 2.1 to 5.1% and the minimal detectable change from 8.6% to 17.8%.

Peak isometric hip torque (Nm/kg): Assessor 1 intra-tester, inter-day reliability

		Test		Retest		Difference test-retest		ICC (CI 95%)	SEM	% SEM	MDC	% MDC
		Mean	SD	Mean	SD	Mean	SD					
ABD	DOM	1.93	0.28	2.02	0.27	0.09	0.14	0.93 (0.76-0.98)	0.11	3.61	0.29	14.89
	NDOM	1.90	0.21	1.92	0.29	0.03	0.16	0.88 (0.64-0.93)	0.11	3.83	0.30	15.87
ADD	DOM	2.14	0.26	2.21	0.29	0.07	0.14	0.86 (0.55-0.95)	0.10	3.01	0.27	12.46
	NDOM	2.04	0.28	2.21	0.35	0.17	0.15	0.87 (0.58-0.96)	0.13	4.26	0.37	17.50
FLEX	DOM	1.21	0.21	1.26	0.20	0.05	0.04	0.98 (0.94-0.99)	0.04	2.08	0.11	8.60
	NDOM	1.23	0.24	1.29	0.24	0.06	0.06	0.97 (0.91-0.99)	0.05	2.80	0.15	11.53
EXT	DOM	1.70	0.47	1.77	0.47	0.07	0.09	0.98 (0.96-0.99)	0.07	2.65	0.19	10.93
	NDOM	1.68	0.46	1.81	0.48	0.13	0.08	0.99 (0.98-1.0)	0.09	3.60	0.26	14.79
IR	DOM	0.81	0.14	0.84	0.14	0.03	0.08	0.79 (0.35-0.93)	0.05	4.25	0.14	17.57
	NDOM	0.75	0.14	0.79	0.15	0.03	0.05	0.92 (0.75-0.97)	0.04	3.45	0.11	14.26
ER	DOM	0.63	0.11	0.63	0.17	0.00	0.06	0.67 (0.035-0.89)	0.04	4.27	0.11	17.75
	NDOM	0.61	0.07	0.64	0.10	0.03	0.07	0.82 (0.45-0.94)	0.05	5.13	0.13	21.16

ABD – abduction, ADD – adduction, FLEX – flexion, EXT – extension, IR – internal rotation, ER – external rotation, DOM – dominant limb, NDOM – non-dominant limb, ICC – interclass correlation coefficient, SEM – standard error of measure, MDC – minimal detectable change

<u>Appendix A2:</u>

Double-leg and single-leg drop jump protocol

Athletes stepped off a 30cm (DLDJ) or 20cm (SLDJ) box and were instructed to keep hands on hips, spend minimal time on the ground and jump vertically as high as possible. Three practice and three maximum effort trials were performed. Jumps were separated by a 30-second rest period and two-minute rest between DLDJs and SLDJs. DLDJs were performed first followed by SLDJs on the non-symptomatic (AGP) or dominant (CON) side.

Appendix A3:

Intersegment control rehabilitation program

The rehabilitation program was designed to address trunk, pelvis and lower limb intersegmental control through strengthening, linear running and change-of-direction mechanics (King *et al.*, 2018). Intersegmental control describes the relationship between the trunk, pelvis, hip, knee and ankle during dynamic movements and a loss of control has been suggested to play a role in the propagation of athletic groin pain. (Franklyn-Miller *et al.*, 2017).

The intersegmental control rehabilitation program consists of three levels, with each level designed to address specific components of recovery. Level 1 focuses on intersegmental control through strengthening exercises, level 2 focuses on intersegmental control through linear running technique and progression of running load tolerance, and level 3 focuses on intersegmental control through change-of-direction technique and progression back to high speed running. Progression from level 1 to level 2 is achieved with a pain-free cross-over test (in the modified Thomas test position), progression from level 2 to 3 occurs with pain-free squeeze test (in 45° hip flexion), pain-free completion of linear 'Run A' program and symmetrical hip range-of-motion (flexion 90° internal rotation mobility). Finally, progression from level 3 to return-to-play is achieved with pain-free completion of 'Run B' program and multi-directional drills. Table 1 outlines the exercise streams for each level of the program and Table 2 outlines the exercise progressions for each of the exercise streams.

Once athletes are cleared to return-to-play, they are advised to complete one full strength session per week (i.e. level 1 control, strength and power exercises), three sessions of the level 1 intersegmental control exercises (e.g. as part of an athlete's pre-pitch/gym preparation) and are to perform the linear and multi-directional technique drills prior to their sporting activity.

Table 1: Outline of exercise streams prescribed during each level of the intersegmental rehabilitation program

**LEVEL 1**	***Stream***	***Segment***	***Target***	***set***	***reps***
Control	Hip flexor holds	Pelvis on femur	Isometric inner range hip flexion	3	8
	Palloff press*	Thorax on pelvis/pelvis on femur	Unilateral obliques	3	8
	Deadbugs	Thorax on pelvis/pelvis on femur	Bilateral obliques	3	8
	Hip hitch	Pelvis on femur	Inner range hip abduction	3	8
	Banded turn outs	Pelvis on femur	Hip external rotation / abduction	3	8
Strength	Single leg hip thrust*	Thorax on pelvis/pelvis on femur	Single leg hip extension	3	8
	Front squat	Multi-segmental	Front squat	3	8
	Deadlift	Multi-segmental	Deadlift	3	8
	Split squat	Multi-segmental	Split Squat	3	8
Power	Ankling	Multi-segmental	Reactive strength	4	10
**Progression criteria - pain-free crossover test**					
**LEVEL 2**	*Completed prior to Run A and B*				
	Switch step	Multi-segmental	Lateral hip RFD	3	3
	Vertical skips	Multi-segmental	Vertical plyometric	4	20
	Resisted box lunge *	Multi-segmental	Horizontal RFD	3	3
**Progression criteria - symmetrical hip flexion (90°)/ IR range, pain-free squeeze (45° hip flexion) and completion Run A program**					
**LEVEL 3**	*Completed prior to Run B*				
	Lateral jump/land*	Multi-segmental	Lateral deceleration RFD	3	3
	Side shuffle cut	Multi-segmental	Lateral reactive strength	3	3
	180° cut	Multi-segmental	RFD + deceleration/reacceleration	3	3
**RTP Criteria – pain-free completion Run B program and multi-directional drills**					

** Exercise additions to the rehabilitation program from King et al. (2018), RFD – rate of force development, IR – internal rotation, RTP – return to play*

Table 2: Progressions for each exercise stream during each level of the intersegmental rehabilitation program: 1 – basic level, 2 – intermediate (entry) level, 3 – advanced level

**LEVEL 1**	***Stream***	***1***	***2***	***3***
Control	Hip flexor holds	Supine	Supported stand	Unsupported stand
	Palloff press	Half kneel	Stand	Stand + rotation
	Deadbugs	Crook lie leg lifts	Crook lie alternate leg lowers	Crook lie bilateral leg lowers
	Hip hitch	Supported stand	Unsupported stand	Low box step-up
	Banded turn outs	Light resistance	Moderate resistance	Heavy resistance
Strength	Single leg hip thrust	Supported	Unsupported	Loaded
	Front squat	High box (60cm)	Low box (45cm) (20% BW)	Low box loaded
	Deadlift	Hip hinge	Rack deadlift loaded (40% BW)	Rack deadlift loaded
	Split squat	Supported	Bodyweight	Loaded
Power	Ankling	Supported single taps	Unsupported single tap	Unsupported double taps
**LEVEL 2**				
	Switch step	March step	Switch step with 5kg plate	Overhead press 5kg plate
	Vertical skips	Overhead march	Vertical skip 50% intensity	Build intensity +/- load
	Resisted box lunge ƚ	Unresisted lunge	Resisted lunge single band	Resisted lunge double band
**LEVEL 3**				
	Lateral jump/land ƚ	0.5-meter jump/land	1-meter jump/land	1-meter + 5kg medball
	Side shuffle cut	50% intensity	Build intensity	Shadow opponent
	180° cut	50% intensity	Build intensity	5kg medball

ƚ Exercise modifications to the rehabilitation program from King et al. (2018), BW – bodyweight

Equipment

The equipment required includes a squat rack, barbell, weights, box/chair (60cm, 45cm), resistance bands and 5kg medicine ball.

Rehabilitation sessions

Rehabilitation sessions were taken by one of three physiotherapists who were trained in the delivery of the intersegmental control rehabilitation program. All physiotherapists had five or more years clinical experience.

Athletes started their rehabilitation on the same day as their initial testing session. Each rehabilitation session was one-to-one and approximately 1 hour in length. Athletes are taken through the various exercises depending on which rehabilitation level they are in (Table 1) with the difficulty of the exercise selected, progressed or regressed depending on the participants ability to execute with appropriate technique (good form and no reproduction of symptoms) (Table 2). These sessions took place every 14-21 days depending on athlete availability.

In between supervised rehabilitation sessions, athletes were instructed to perform the rehabilitation program at their own training base. Each subject was given a printed handout of all the exercises with the main coaching cues for each), in addition to each exercise being captured on video using Dartfish™ software and the videos hosted online for the athlete to review between sessions. Athletes were instructed to complete a weekly exercise diary to help ensure compliance. Athletes were advised that should any exercise reproduce their symptoms, they should review their videos and amend their technique to resolve and if unable to do so these exercises should be discontinued and their physiotherapist will review during the next rehabilitation session.

Level 1

Level 1 exercises focus on both the control between two segments across one joint (e.g. hip flexor holds) and also multi-segmental movements (e.g. deadlift). For the loaded exercises, the deadlift weight started at approximately 40% bodyweight, front squat 20% bodyweight and split squat bodyweight only. The weight was gradually increased as athletes could reach the appropriate number of repetitions in each set with good control and no symptoms. The importance of correct technique was repeatedly emphasized. Level 1 exercises were included throughout the duration of the rehabilitation program.

Level 2

The focus of the level 2 exercise streams (Table 1) is to improve intersegmental control during linear running with exercise drills designed to address commonly observed technical faults (e.g. poor lumbopelvic control at toe-off, poor swing leg recovery) or reduced neuromuscular capacity (e.g. contralateral hip drop during the stance phase of the running gait). Once athletes complete the linear running drills, they carry out the Run A program (Table 3). This was developed for field-sport athletes to gradually build tolerance to running loads and intensity while assessing the athletes' tolerance and suitability for progression. It starts with low volume and low intensity, both of which increase at different points through the program. Athletes are instructed to progress, or regress based on a traffic-light system. Green light means no pain during or the following day and athletes can progress to the next running session. Red light means increased pain during or the following day and athletes are instructed to repeat the same session or drop back a session when scheduled until they can tolerate it and then progress to the next session (by obtaining a green light).

Table 3: Run A and B programs included in Level 2and 3 of the intersegmental control rehabilitation and comparison to King et al (2018).

Baida et al (2020)

**Run A (Level 2)**		**Distance (m)**	**Time (sec)**	**Speed (km/h)**	**sets**	**reps**	**Work:Rest ratio**	**Speed Distance (m)**	**Total Distance (m)**
Warm-up		400		50%	1	2	1:1	0	800
Linear exercise drills									
session 1	Intervals	20/80/20	24	12	2	8	1:2	1920	2720
session 2		20/80/20	24	12	2	10	1:2	2400	3200
session 3		20/80/20	21	14	2	10	1:2	2400	3200
session 4		20/80/20	21	14	3	8	1:2	2800	3400
session 5		20/80/20	19	16	3	8	1:2	2880	3480
session 6		20/80/20	17	18	3	8	1:2	2880	3480
**RUN B (Level 3)**									
Warm-up		400		50%	1	2	1:1	0	800
Linear exercise drills									
Multi-directional exercise drills									
session 1	Sprints (rolling start)	30	7	18	3	2	1:10	180	
	Intervals	80	25-22	14-16	6	3	1:1	1440	2420
session 2	Sprints (rolling start)	30	6	20-22	3	2	1:10	180	
	Intervals	80	25-22	14-16	6	3	1:1	1440	2420
session 3	Sprints (rolling start)	30	6	20-22	5	2	1:10	300	
	Intervals	80	25-22	14-16	6	3	1:1	1440	2540
session 4	Sprints (standing start)	30	6	20-22	5	2	1:10	300	
	Intervals	80	25-22	14-16	6	4	1:1	1920	3020
session 5	Sprints (rolling start)	30	5	24-26	5	2	1:10	300	
	Intervals	80	25-22	14-16	6	4	1:1	1920	3020
session 6	Sprints (standing start)	30	5	24-26	5	2	1:10	300	
	Intervals	80	25-22	14-16	6	4	1:1	1920	3020

King et al (2018)

**Run A (Level 2)**		**Distance**	**Speed**	**set**	**rep**	**Rest (rep)**	**Distance**	**Total Distance**
Linear exercise drills								
session 1	Intervals	400	50%	1	6	60sec	2400	2400
session 2		400	50%	1	8	60sec	3200	3200
session 3		400	50%	1	10	60sec	4000	4000
session 4		400	70%	1	10	60sec	4000	4000
session 5		400	85%	1	10	60sec	4000	4000
session 6		400	100%	1	10	60sec	4000	4000
**RUN B (Level 3)**								
warm-up		400	70%	1	4	60sec	1600	
Linear exercise drills								
Multi-directional exercise drills								
session 1	Sprints (rolling start)	100	70%	1	10	60sec	1000	2600
session 2	Sprints (rolling start)	100	85%	1	10	30sec	1000	2600
session 3	Sprints (rolling start)	100	100%	1	10	30sec	1000	2600
session 4	Sprints (rolling start)	100	100%	1	5	30sec	500	
	Sprints (standing start)	50	70%	1	5	30sec	250	2350
session 5	Sprints (rolling start)	100	100%	1	5	30sec	500	
	Sprints (standing start)	50	85%	1	5	30sec	250	2350
session 6	Sprints (rolling start)	100	100%	1	5	30sec	500	
	Sprints (standing start)	50	100%	1	5	30sec	250	2350
session 7	Sprints (rolling start)	100	100%	1	5	30sec	500	
	Sprints (standing start)	50	100%	1	10	30sec	500	2600

Level 3

The focus of the level 3 exercise streams is to improve intersegmental control during multidirectional movements, lateral rate of force development and agility prior to returning to sports (Table 1). Athletes also progress from Run A to Run B program which increases exposure to high speed running. Once athletes complete the multidirectional drills (and linear run drills) they carry out the Run B program. The drills are executed at as high an intensity as possible without reproduction of symptoms.

Weekly frequency

Level 1 of the rehabilitation program is prescribed on a 3-day cycle: day 1 all exercises (control, strength and power), day 2 control exercises only and day 3 rest. Level 2 and 3 is prescribed on a 4-day cycle: day 1 all exercises (control, strength and power), day 2 Run A or B (depending on which level an athlete is at), day 3 control exercises only, day 4 rest.

Appendix A4:

Outcome measures: Peak isometric hip torque, hip torque ratios, double-leg and single-leg reactive strength and bilateral

	AGP pre	AGP post	CON	AGP pre vs. CON				AGP pre vs. AGP post				AGP post vs. CON			
Peak torque (Nm/kg)	Mean ±SD	Mean ±SD	Mean ±SD	p	*d*	% Diff	Cons (%)	p	*d*	% Δ	Cons (%)	p	*d*	% Diff	Cons (%)
ABD	1.84 ± 0.31	2.11 ± 0.38	2.28 ± 0.27	0.000*	-1.20	24%	100	0.000*	-0.83	15%	100	0.066	-0.58	8%	60
ADD	2.18 ± 0.43	2.73 ± 0.35	2.81 ± 0.39	0.000*	-1.20	29%	100	0.000*	-1.15	25%	100	0.505	-0.20	3%	3
FLEX	1.18 ± 0.21	1.36 ± 0.17	1.43 ± 0.19	0.000*	-1.07	22%	100	0.000*	-0.86	15%	100	0.212	-0.40	6%	19
EXT	1.83 ± 0.43	2.33 ± 0.37	2.20 ± 0.39	0.005*	-0.83	21%	100	0.000*	-1.05	27%	100	0.303	0.32	-5%	4
ER	0.65 ± 0.14	0.78 ± 0.11	0.75 ± 0.14	0.030*	-0.67	15%	85	0.000*	-0.92	20%	100	0.394	0.26	-4%	4
IR	0.91 ± 0.18	1.06 ± 0.16	0.95 ± 0.18	0.439	-0.23	5%	1	0.000*	-0.84	17%	100	0.035	0.64	-11%	73
**Torque ratio**															
ADD/ABD	1.21 ± 0.26	1.31 ± 0.21	1.25 ± 0.21	0.556	-0.16	3%	0	0.041	-0.43	9%	75	0.328	0.31	-5%	10
EXT/FLEX	1.57 ± 0.30	1.73 ± 0.24	1.55 ± 0.22	0.638	0.07	-1%	0	0.009*	-0.55	10%	99	0.018*	0.71	-10%	90
ER/IR	0.72 ± 0.14	0.76 ± 0.13	0.80 ± 0.13	0.061	-0.58	11%	60	0.222	-0.25	5%	8	0.254	-0.37	6%	14
**Reactive strength**															
DLDJ GCT (sec)	0.30 ± 0.08	0.26 ± 0.06	0.26 ± 0.09	0.158	0.42	-12%	15	0.010*	0.54	-13%	100	0.726	-0.03	1%	0
DLDJ JH (cm)	30.49 ± 5.30	29.70 ± 5.12	30.59 ± 3.93	0.672	-0.03	0%	0	0.373	0.15	-2%	2	0.507	-0.20	2%	0
DLDJ RSI	1.09 ± 0.33	1.19 ± 0.30	1.29 ± 0.40	0.082	-0.53	18%	49	0.086	-0.32	9%	41	0.366	-0.27	8%	2
SLDJ GCT (sec)	0.37 ± 0.07	0.32 ± 0.04	0.33 ± 0.06	0.050	0.58	-11%	64	0.001*	0.76	-14%	100	0.555	-0.17	3%	0
SLDJ JH (cm)	13.42 ± 3.08	13.15 ± 3.18	15.21 ± 2.45	0.050	-0.61	13%	68	0.542	0.09	-2%	0	0.029	-0.68	16%	83
SLDJ RSI	0.38 ± 0.13	0.42 ± 0.12	0.48 ± 0.12	0.014*	-0.73	26%	97	0.093	-0.30	9%	45	0.099	-0.51	15%	43
**Squeeze test (mm Hg)**															
SQ0 Max	151 ± 24	170 ± 28	171 ± 32	0.024*	-0.66	13%	86	0.008*	-0.66	12%	100	0.662	-0.05	1%	0
SQ0 P1	126 ± 17	N/A	N/A	N/A	N/A	N/A	N/A	N/A	N/A	N/A	N/A	N/A	N/A	N/A	N/A
SQ45 Max	257 ± 38	276 ± 27	273 ± 39	0.177	-0.41	6%	19	0.006*	-0.57	8%	100	0.627	0.09	-1%	0
SQ45 P1	128 ± 36	N/A	N/A	N/A	N/A	N/A	N/A	N/A	N/A	N/A	N/A	N/A	N/A	N/A	N/A

*ABD – abduction, ADD – adduction, FLEX – flexion, EXT – extension, ER – external rotation, IR –internal rotation, % Diff – percent mean difference, % Δ - percent change in mean score, cons – consistency of p<0.05, DL – double leg, GCT – ground contact time, JH – jump height, RSI – reactive strength index, SL – single leg, *significant difference (p < 0.05, freq > 85%), SQ45/0 – bilateral squeeze test in 45°/0° hip flexion, MAX – maximum value, P1 – value recorded at onset of pain occurred, N/A – not applicable (no squeeze pain reported in AGP post or CON g*

Appendix A5:

Asymmetry index of peak isometric hip torque and single-leg reactive strength

	AGP pre		AGP post		CON	AGP pre V CON Absolute			AGP pre V AGP post Directional			AGP post V CON Absolute		
**Peak torque (Nm/kg)**	Absolute mean ±SD	Directional mean ±SD	Absolute mean ±SD	Directional mean ±SD	Absolute mean ±SD	p	*d*	Cons (%)	p	*d*	Cons (%)	p	*d*	Cons (%)
ABD	8.62 ± 7.69	-0.15 ± 11.50	7.05 ± 5.67	1.48 ± 8.89	8.56 ± 5.13	0.239	0.01	6	0.400	-0.16	1	0.133	-0.27	20
ADD	9.94 ± 8.86	-4.21 ± 12.58	6.23 ± 4.78	0.77 ± 7.80	7.91 ± 5.39	0.328	0.27	0	0.115	-0.46	30	0.148	-0.33	16
ER	12.42 ± 9.07	-3.22 ± 15.01	10.52 ± 6.10	-1.03 ± 12.06	7.90 ± 5.67	0.049	0.57	64	0.450	-0.16	0	0.076	0.43	42
EXT	12.31 ± 8.96	-0.31 ± 15.17	8.54 ±5 .65	3.64 ± 9.54	11.29 ± 8.04	0.338	0.12	0	0.171	-0.31	22	0.180	-0.39	10
FLEX	9.32 ± 6.65	-3.02 ± 11.01	5.61 ± 4.86	-1.77 ± 7.18	6.18 ± 4.22	0.059	0.54	55	0.565	-0.13	1	0.269	-0.13	2
IR	13.00 ± 8.89	-3.3 ± 15.33	11.02 ± 9.46	-3.52 ± 14.05	9.85 ± 6.74	0.142	0.39	14	0.727	0.01	0	0.330	0.14	1
**Reactive strength**														
SLDJ CT (sec)	9.47 ±7.50	2.50 ±11.77	6.79 ±5.88	2.59 ±8.58	5.98 ±5.13	0.057	0.52	61	0.698	-0.01	0	0.297	0.15	0
SLDJ JH (cm)	10.77 ±9.61	-3.55 ±13.93	14.85 ±16.28	-3.05 ±21.69	10.57 ±7.89	0.273	0.02	5	0.687	-0.02	0	0.278	0.32	3
SLDJ RSI	15.18 ±14.38	-5.49 ±20.11	15.42 ±14.96	-5.39 ±20.66	11.64 ±10.00	0.257	0.28	4	0.695	-0.01	0	0.212	0.29	4

ABD – abduction, ADD – adduction, FLEX – flexion, EXT – extension, ER – external rotation, IR –internal rotation, SL – single leg, GCT – ground contact time, JH – jump height, RSI – reactive strength index, cons – consistency of p<0.05.

Nb: a negative sign in the directional asymmetry value indicates a greater value on the non-symptomatic limb
